# Long-Term Retention of Avulsed Maxillary Incisors with Replacement Root Resorption: A 9-Year Follow-Up

**DOI:** 10.1155/2021/8872859

**Published:** 2021-01-11

**Authors:** Hyunjung Yoon, Minju Song

**Affiliations:** Department of Conservative Dentistry, College of Dentistry, Dankook University, Cheonan, Republic of Korea

## Abstract

The purpose of this case report is to present a case of long-term retention of avulsed upper maxillary incisors with external replacement root resorption in a 15-year-old boy. The avulsed teeth, #11, 21, and 22, were stored under dry conditions for 40 min and replanted. Endodontic treatment was initiated after two weeks, and the nonrigid splint was removed after 3.5 months. A year after replantation of the teeth, replacement root resorption was detected radiographically. For the following 9 years, the resorption progressed slowly, but the teeth were maintained without any adverse effects on esthetic appearance. Under dry storage conditions, replacement root resorption was expected. In this case, the patient was a growing 15-year-old boy; thus, replantation was performed despite a possible poor prognosis. Consequently, root resorption progressed. Nevertheless, maintenance of the tooth crowns led to satisfying results for the patient both esthetically and psychologically. Ankylosis or replacement root resorption is a complication occurring after replantation of avulsed teeth, which could require additional treatment. However, in young patients, replantation could be considered to maintain the teeth until growth is complete.

## 1. Introduction

The prevalence of avulsion of permanent teeth is reported to be 0.5 to 3.0%. Avulsion is most common in young permanent dentition [1, 2]. This is because in childhood, the root is not yet completely formed and the alveolar bone is very resilient [1]. Compared with other types of dental injuries, prompt emergency treatment for the avulsed tooth is critical to prevent complications relating to the pulp and the periodontal ligament.

For the avulsed permanent teeth, pulp necrosis is inevitable because the nerves and blood vessels of the injured teeth have been severed. However, timely root canal therapy, initiated within 10 days, and placement of Ca(OH)_2_ as intracanal medication could prevent inflammatory external root resorption derived from the necrotic pulp [3, 4]. To prevent replacement root resorption or ankylosis caused by damaged periodontal ligament tissues, physiologic storage medium and functional splinting are required. Replacement root resorption cannot be controlled once initiated; therefore, prevention by minimizing damage to the periodontal ligament is the only treatment [5].

Clinically, teeth with replacement root resorption present with a high-pitched metallic percussion sound and no physiological mobility [6, 7]. Radiographically, teeth with replacement root resorption show a lack of periodontal ligament space and unclear outlines of the roots. In adults, replacement root resorption may be considered an acceptable outcome. However, in young, growing patients, progression of replacement resorption could lead to tooth loss and ankylosis could result in infraposition of teeth, which would require further treatments such as surgical procedure, orthodontic treatment, and/or tooth extraction [8]. Therefore, the management of avulsed teeth in growing patients is complicated. In cases where negative long-term prognosis is expected, leaving the socket empty without replantation may be considered appropriate.

This case report presents the long-term retention of avulsed maxillary incisors with replacement root resorption in a 15-year-old patient. Although there was progression of replacement root resorption, the patient's teeth were maintained without any adverse effects esthetically, during a 9-year follow-up period.

## 2. Case Presentation

**Figure 1:**
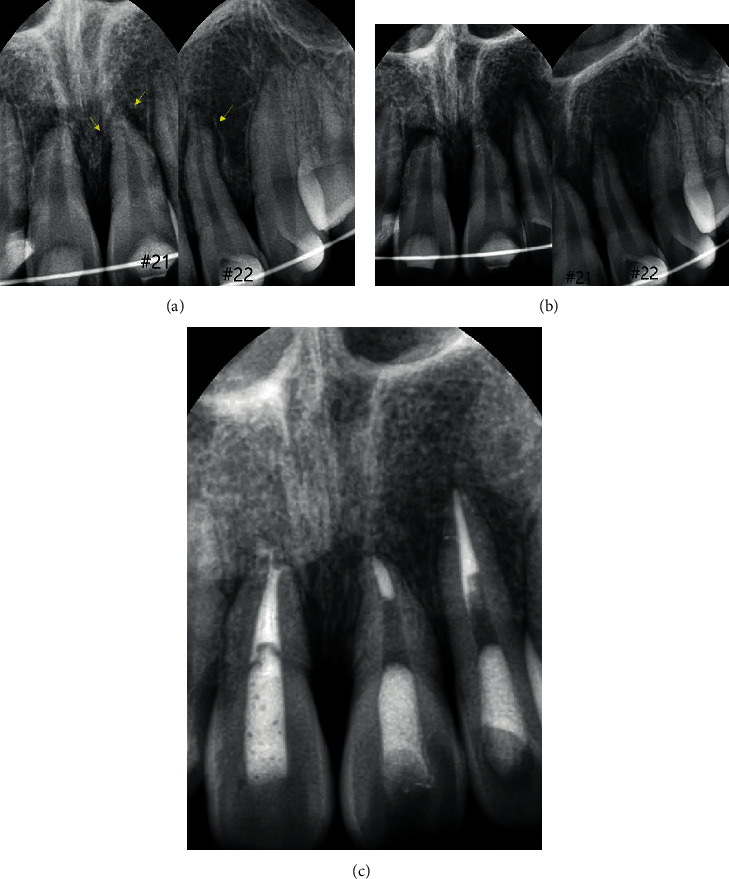
(a) Splinting of the avulsed teeth after replantation. The space around the roots (yellow arrows) indicates improper repositioning. (b) Periapical radiographs two weeks after traumatic injury. (c) Endodontic treatment of the avulsed teeth all completed 14 weeks after the trauma.

A 15-year-old boy with traumatic injury to his maxillary incisors was referred to the Department of Conservative Dentistry, Dental Hospital of Dankook University. He had a bicycle accident a day ago and received emergency treatment for his injured anterior teeth at the emergency room of Dankook University Hospital. The patient's medical history and extraoral findings were unremarkable. His maxillary anterior teeth were splinted with composite and a 0.18-inch stainless steel wire, from the left first premolar to the right first premolar (#14 to #24). Intraoral examination revealed tenderness upon percussion of the maxillary anterior teeth and an uncomplicated crown fracture on the maxillary right lateral incisor. On the periapical radiographs, clear radiolucency between the root surface and alveolar socket, suggestive of improper repositioning, was detected in teeth #21 and 22 (Figure 1(a)). As more than 24 h had passed since the accident, repositioning was not attempted.

According to the record of the emergency room and patient interview, both maxillary central incisors (#11 and 21) and the maxillary left lateral incisor (#22) were avulsed. Alveolar bone fracture of the anterior maxillary was also noted. The fractured labial bone plate was separated from the attached gingiva, which was lacerated. The interdental papillae were also torn. First aid was performed as follows. The two avulsed teeth (#21 and 22) were stored under dry conditions for more than 40 min. Another avulsed tooth (#11) was brought in by the patient's friend later. All the avulsed teeth were contaminated with dirt and were thus rinsed with saline before replantation. Other root surface treatment was not preformed. Replantation of the teeth was performed under local anesthesia (2% lidocaine) with slight finger pressure. Appropriate repositioning was difficult because of the alveolar bone fracture. As there was no dental intraoral X-ray system at the emergency room, correct positioning of the teeth was not guaranteed. Instead, positioning was confirmed by the patient and his friend. A flexible splint was applied from teeth #14 to #24 using wire and composite resins. Systemic antibiotic therapy was administered.

Two weeks after tooth injury, an oral examination was performed (Figure 1(b)). The avulsed teeth #11, 21, and 22 did not respond to electric pulp testing (Parkell Inc., NY, USA) and revealed an uncertain reaction to the thermal sensibility test. All the teeth were tender upon percussion. Root canal treatment was performed on the avulsed teeth. Under rubber dam isolation, the necrotic pulp tissue was removed, and root canals were chemomechanically prepared. Sodium hypochlorite (2.5%) was used as an essential irrigant, and calcium hydroxide (Ultracal®; Ultradent Inc., South Jordan, USA) was used for intracanal medication. After five weeks of intracanal dressing, teeth #21 and 22 were obturated using the lateral compaction technique with gutta-percha and epoxy resin-based root canal sealer (AH-26 sealer, Dentsply, Konstanz, Germany). Removal of the splint was scheduled for the next visit, but the patient unfortunately failed to attend his appointment. Thus, splinting was removed 14 weeks after the accident, and tooth #11 was obturated. The access cavities of teeth #11, 21, and 22 were filled with Fuji II LC (GC, Tokyo, Japan) and Z350 composite resin (3M ESPE, St. Paul, MN, USA).

**Figure 2:**
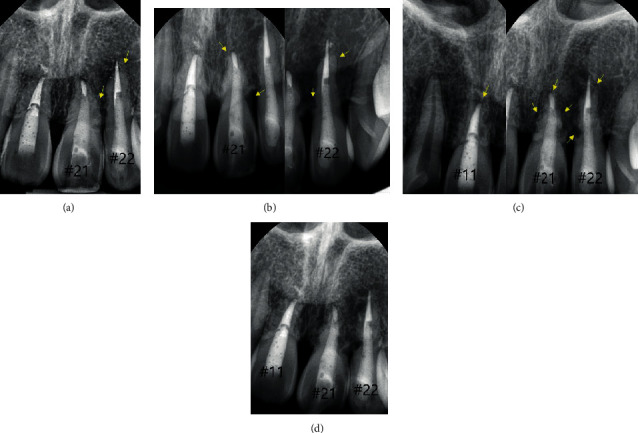
(a) Follow-up after 1 year with radiological signs of external replacement resorption. External root resorption in the distal part of tooth #21 and apical part of tooth #22. Gutta-percha extrusion in tooth #22 and a slightly moth-eaten appearance at the mesial surface of tooth #21. (b) Follow-up after 2 years with ongoing replacement resorption in teeth #21 and 22 (yellow arrows) (c) Follow-up after 3 years. External root resorption in the entire surface of tooth #11 for the first time (moth-eaten appearance). Progression of replacement resorption in teeth #21 and 22 (yellow arrow). (d) Follow-up after 4 years with slow progression of external replacement resorption (teeth #11, 21, and 22).

**Figure 3:**
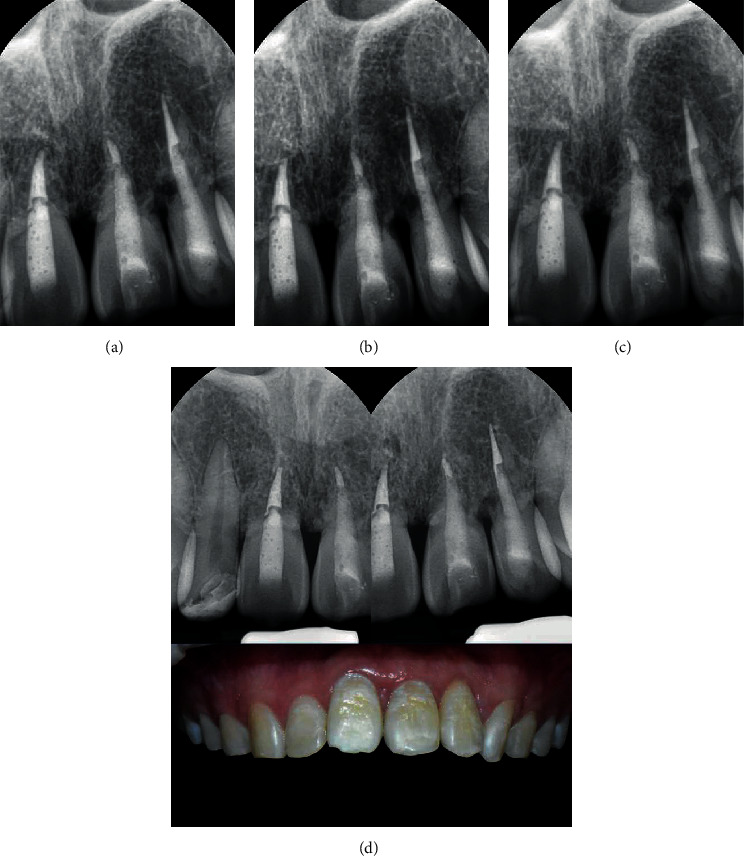
Periapical radiographs show gradual progression of root resorption. At the 9-year follow-up, clinical photograph of the avulsed teeth was also taken. ((a) 4 years: 10 m; (b) 5 years: 7 m; (c) 6 years: 2 m; (d) 9 years).

The 1-year follow-up revealed radiological signs of external root resorption in the patient's maxillary left incisors (#21 and 22) but not in tooth #11 (Figure 2(a)). All the teeth were asymptomatic. At the 2-year follow-up, clinical and radiological examinations revealed progression of external root resorption of teeth #21 and 22 (Figure 2(b)). The patient was informed that root resorption had progressed, and further treatment such as extraction and dental implant may be required. At the 3-year follow-up, tooth #11 showed clear radiological signs of external root resorption (Figure 2(c)). Replacement root resorption also seemingly progressed up to the 4-year, 4.8-year, and 5.6-year follow-up for tooth #22, 21, and 11, respectively. However, resorption seemed to slow down at the yearly visit thereafter (Figure 3). At the 9-year follow-up, which was the latest visit of the patient, a stable, aesthetically pleasing state of the teeth with no pathological findings or unpleasing problems was noted (Figure 3(d)). Further yearly recall visits were scheduled to monitor the progress of the resorption.

## 3. Discussion

This case report presents long-term progression of replacement root resorption. Replacement root resorption is one of the complications of avulsed teeth. Since root canal therapy was initiated two weeks after trauma, external inflammatory resorption resulting from an infection of the pulpal space was unlikely in this case. However, necrosis of the periodontal ligament cells was unavoidable. According to the record, the patient arrived at emergency room within 60 min after trauma, but his teeth were brought in, in a dry condition. In addition, the spaces between the alveolar sockets and root surfaces of teeth #21 and 22 were clearly shown on the periapical radiographs taken the next day, indicating improper repositioning. Based on these observations, it was possible to predict that replacement resorption would occur as a complication.

In general, root dentin is protected by organic tissues namely, the cementum and healthy periodontal ligament. However, when the periodontal apparatus is damaged during trauma, osteoclasts move to the exposed dentin surface and begin to resorb inorganic dental tissues [5, 9]. In cases where the exposure site is small, for example, less than 20% of the root surface, resorption can be self-limiting and clinically insignificant [5, 10]. Otherwise, root resorption progresses and replacement with generated bone tissue occurs. Clinical signs of root resorption include loss of physiologic mobility and a high-pitched metallic sound upon percussion [8, 11], a phenomenon termed as “dento-alveolar ankylosis” [5]. The resorption of root dentin by osteoclasts and its replacement with bone tissues by osteoblasts are part of the same repair process as that of physiologic bone remodeling. Therefore, like bone remodeling, it progresses without the need for any specific stimulation and at a similar speed [12, 13].

The primary factor influencing the progress of replacement resorption is age [14–16]. Being of the age around which the growth spurt takes place affects the prognosis of replanted teeth, as such patients have a rapid rate of bone remodeling [17]. Therefore, replacement root resorption will progress in a rapid manner. The root will completely resorb, and the patient will eventually end up with tooth loss. Most studies report that replacement root resorption is usually detected within one to two years after avulsion and that the replanted ankylotic teeth are usually lost within four to six years [18]. Andersson et al. [14] also concluded that replanted teeth will become ankylosed and resorbed within three to seven years in patients aged 8–16 years, whereas teeth under same condition may remain functional for a longer period in patients older than 17 years old. They used a radiographic index to assess the replacement resorption and reported that patients aged 8–16 years reached the maximum score of 12 (most severe resorption) within four years, whereas older patients showed a low index of 5–6 at 12.5 years after replantation [14].

There are several case reports supporting this consensus. Krug et al. [19] reported the long-term outcome of delayed replantation of avulsed anterior teeth in a 18.6-year-old patient. The 1.5- and 3-year follow-up revealed signs of replacement root resorption. However, a 16-year follow-up revealed a stable and slowly progressing root resorption and a low Andersson's index score of 6 and 7 [14]. Meanwhile, Cobankara and Ungor [20] reported a case showing complete root resorption and further tooth extraction after delayed replantation. The patient was 15 years old and had teeth #11 and 21 avulsed. Root resorption was initiated after a 2-year follow-up, which then rapidly progressed to extensive root resorption after eight years. The trajectory of this case is similar to ours.

**Figure 4:**
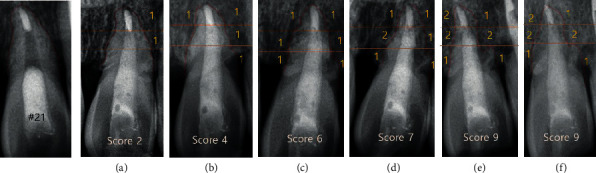
Radiographic index of tooth #21 ((a) 1 year; (b) 2 years; (c) 3 years; (d) 4 years; (e) 4 years: 10 m; (f) 9 years) (Andersson et al.) [14].

**Figure 5:**
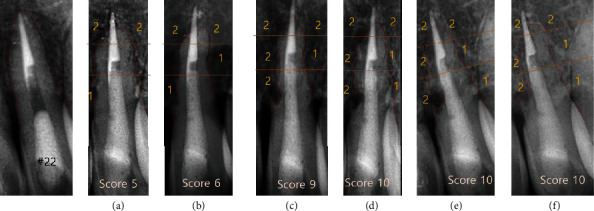
Radiographic index of tooth #22 ((a) 1 year; (b) 2 years; (c) 3 years; (d) 4 years; (e) 4 years: 10 m; (f) 9 years) (Andersson et al.) [14].

**Figure 6:**
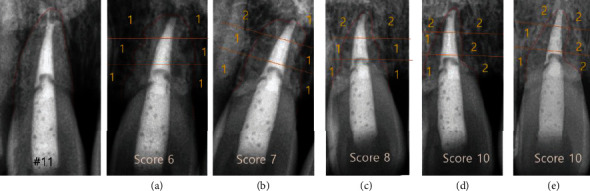
Radiographic index of tooth #11 ((a) 3 years; (b) 4 years; (c) 4 years: 10 m; (d) 5 years: 7 m; (e) 9 years) (Andersson et al.) [14].

In this case, replacement root resorption occurred following the replantation of the patient's avulsed teeth stored in a dry condition. At the 1-year follow-up, root resorption was first radiographically detected on the apical part of tooth #22. It showed a blunt apex with an extruded gutta-percha. At the same time, irregular outlines and radiolucencies were seen on the mesial and distal root surfaces of teeth #21 and 22. It had progressed for three to four years and then seemed to stabilize without any significant change (Figures 4 and 5). Tooth #11 did not show any significant change, whereas root resorption started in teeth #21 and 22. However, at the 3-year follow-up, tooth #11 showed aggressive root resorption, with a score of 6, after which it had progressed for three years and then finally seemed to stabilize like teeth #21 and 22 (Figure 6). Finally, all teeth showed severe resorption with a score of 9–10. Nevertheless, they remained functional for eight years without any aesthetic problem. Furthermore, infraposition of the teeth did not occur. Along with the speed of root resorption, the degree of infraposition also relates to the growth spurt. It has been reported that children under the age of 10 have a high risk of severe infraposition, with this risk decreasing after the peak of growth [21–23].

According to the International Association for Dental Traumatology and the American Association of Endodontists, replantation is recommended even when the avulsed tooth is stored in improper storage media or when dry time is already over 60 min. However, there is poor prognosis and root resorption/ankylosis and infraposition of the tooth can occur [3, 24]. A standard protocol for resorbed and ankylosed teeth has not yet been established. Therefore, many clinicians may prefer not to replant an avulsed tooth with poor prognosis [8]. Nonetheless, tooth replantation is required for the management of avulsed teeth when aesthetical, functional, and psychological aspects are considered, especially for growing patients. In the case of Cobankara and Ungor [20], the teeth remained functional for eight years but were extracted when the teeth showed a pink appearance typically indicating external cervical resorption. Although not mentioned in the report, those teeth would have remained for a longer period if there was no external cervical resorption, which was detected at the patient's 7-year follow-up. There might be concerns about the difficulty of tooth extraction and dental implant replacement for ankylosed teeth. However, there is a report that the installation of implants in the areas with dentoalveolar ankylosis without a complete tooth extraction was no interference with osteointegration [25]. If the resorption would be ongoing, the remaining dentin and periodontal ligament could be replaced with bone totally. Considered the pros and cons of maintenance of the teeth having replacement resorption, maintaining teeth could be considered a priority.

## 4. Conclusion

In this case, replacement root resorption was expected following dry storage of the avulsed teeth. In addition, since the patient was 15 years old and still growing, there might have been acceleration of the progress of the resorption. However, considering the aesthetical, functional, and psychological needs of a growing child, replantation was carried out. Although there was progression of replacement root resorption as expected, the patient's teeth were maintained without occurrence of any adverse changes for nine years. Under most conditions, replantation of avulsed teeth is considered more beneficial than nonreplantation.

## Data Availability

No data were used to support this study.
